# Two-Stage Surgical Treatment for Non-Union of a Shortened Osteoporotic Femur

**DOI:** 10.5812/traumamon.8293

**Published:** 2013-05-26

**Authors:** Galal Zaki Said, Osama Ahmed Farouk, Hatem Galal Said

**Affiliations:** 1 Department of Orthopedic Surgery, Faculty of Medicine, Assiut University Hospitals, Assiut, Egypt

**Keywords:** Nonunion, Femur, Osteoporosis, Bone Lengthening

## Abstract

**Introduction:**

We report a case of non-union with severe shortening of the femur following diaphysectomy for chronic osteomyelitis.

**Case Presentation:**

A boy, aged 16 years presented with a dangling and excessively short left lower limb. He was using an elbow crutch in his right hand to help him walk. He had a history of diaphysectomy for chronic osteomyelitis at the age of 9. Examination revealed a freely mobile non-union of the left femur. The femur was the seat of an 18 cm shortening and a 4 cm defect at the non-union site; the knee joint was ankylosed in extension. The tibia and fibula were 10 cm short. Considering the extensive shortening in the femur and tibia in addition to osteoporosis, he was treated in two stages. In stage I, the femoral non-union was treated by open reduction, internal fixation and iliac bone grafting. The patient was then allowed to walk with full weight bearing in an extension brace for 7 months. In Stage II, equalization of leg length discrepancy (LLD) was achieved by simultaneous distraction of the femur and tibia by unilateral frames. At the 6 month follow- up, he was fully weight bearing without any walking aid, with a heel lift to compensate the 1.5 cm shortening. Three years later he reported that he was satisfied with the result of treatment and was leading a normal life as a university student.

**Conclusions:**

Two-stage treatment succeeded to restore about 20 cm of the femoral shortening in a severely osteoporotic bone. It has also succeeded in reducing the treatment time of the external fixator.

## 1. Introduction

In chronic osteomyelitis of long bones in children, sometimes massive sequestration of the diaphysis occurs. Infection may result in destruction of the proliferative layer of the periostium, defective formation of the involucrum and pathological fracture and/or segmental bone loss. To avoid these possible complications, sequestrectomy is usually delayed until a sufficient involucrum has formed (1).

## 2. Case Presentation

We are reporting a case of non-union of the femur following diaphysectomy for chronic osteomyelitis. The non-union was neglected for 7 years. Non-weight bearing for this prolonged period resulted in severe shortening and osteoporosis. This mandated treatment in two stages; the first stage was open reduction and internal fixation which resulted in healing of the femoral non-union in the shortened position. The second stage was successful correction of the LLD by simultaneous distraction of the femur and tibia.

### 2.1. The Patient

**Figure 1.**
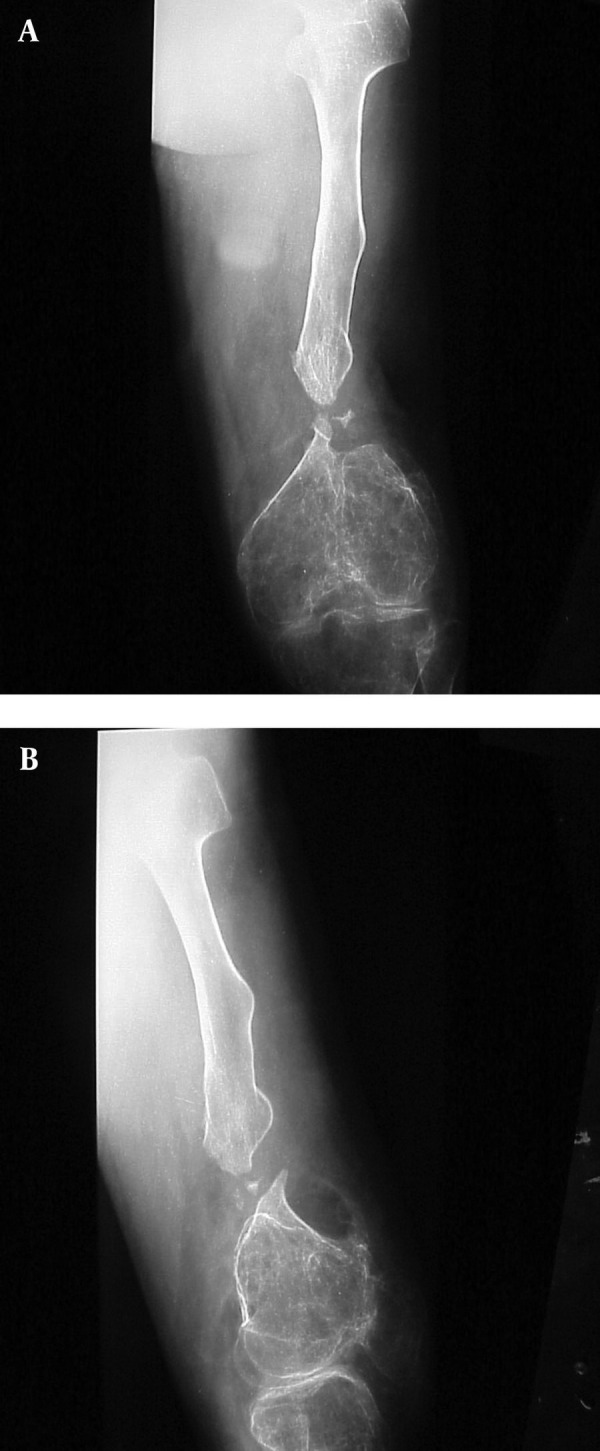
Neglected Non-Union of the Left Femur. A and B) Anterior-Posterior and lateral views of neglected non-union of left femur showing great shortening and severe osteoporosis; There is a bony defect of 4 cm at the non-union site.

A boy from a sub-Saharan African country, aged 16 years was suffering from a dangling non-weight bearing and excessively short left lower limb. He used one elbow crutch with his right hand to aid him in walking. Examination revealed a freely mobile non-union of the left femur, which followed a diaphysectomy for chronic osteomyelitis at age 9. The thigh was firm in consistency and the knee joint was completely stiff in full extension. The left femur was 49.5 cm long, 18 cm shorter than the right femur. The tibia and fibula were 10 cm short. The CBC, ESR and CRP were normal. Radiological examination revealed atrophic non-union of the lower third reject portion of the femur with a 4 cm bony defect. The proximal and distal epiphyseal cartilage plates of the femur, tibia and fibula were closed and the bones were the seats of severe osteoporosis to such a degree that only a ghost of the distal segment of the femur was present. The patella was bony ankylosed to the femur (Figure 1). His quality of life as determined by SF-36v2 PCS was 41.5 and MCS 39.6.

### 2.2. Surgical Techniques and Outcome

**Figure 2.**
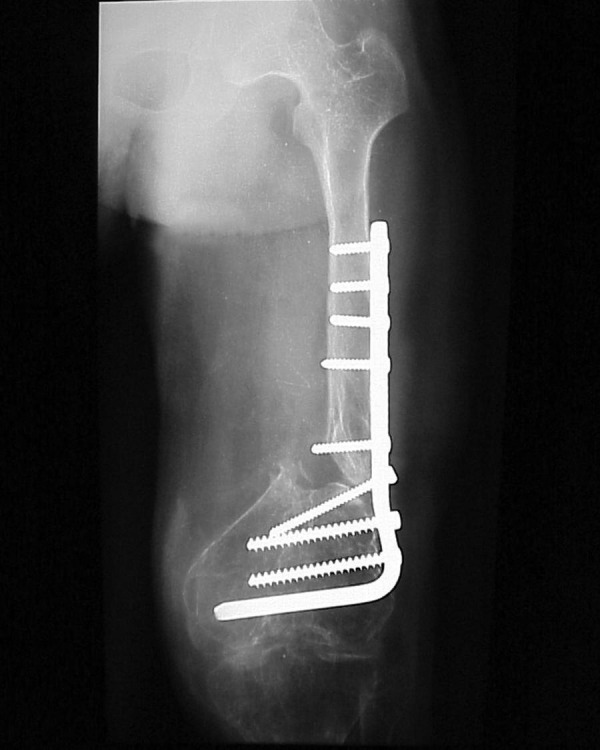
Post-operative Anterior-Posterior Radiograph Showing Approximation of Bone Ends, Condylar Plate Fixation and Iliac Bone Grafting

**Figure 3.**
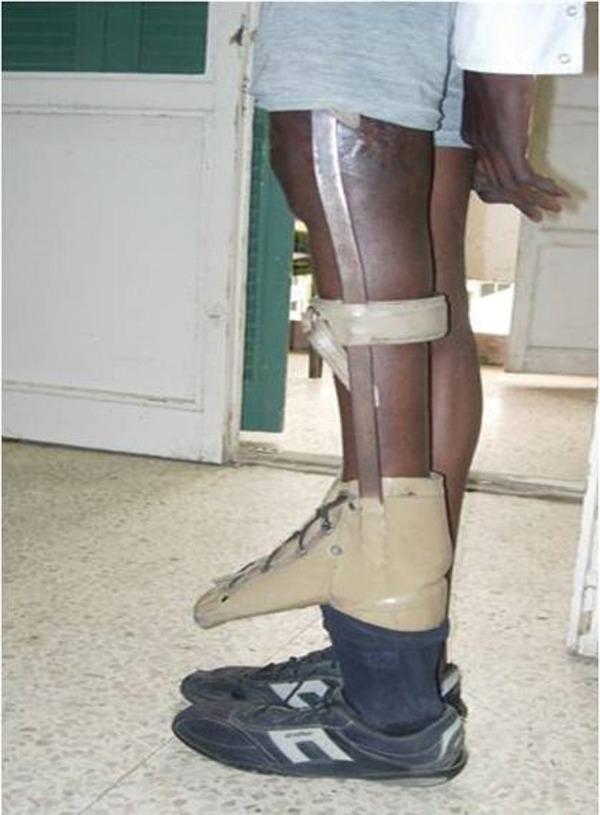
A Photograph at the End of Stage I. Note the short left lower limb in the extension brace; The foot is in the upper boot and the artificial foot is in the lower one.

**Figure 4.**
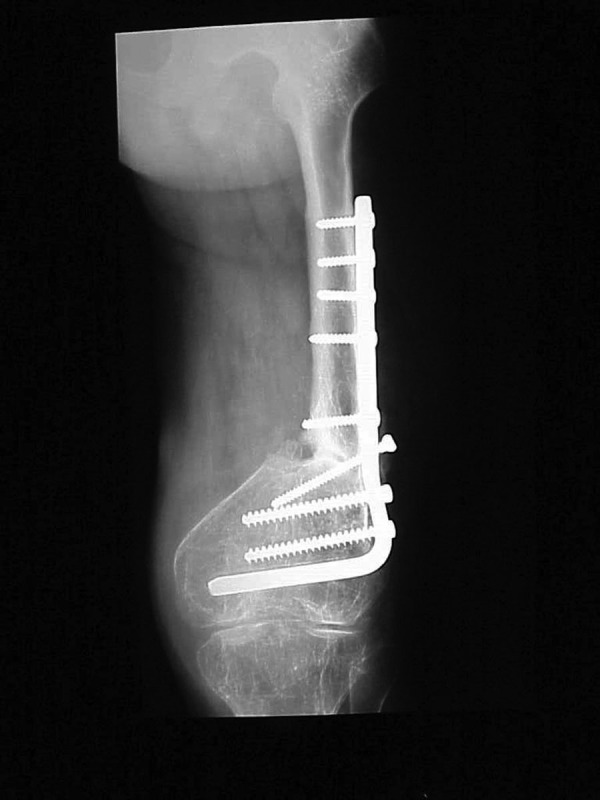
Anterior-posterior View After 7 Months of Weight Bearing in the Extension Brace, Showing Improved Bone Quality

**Figure 5.**
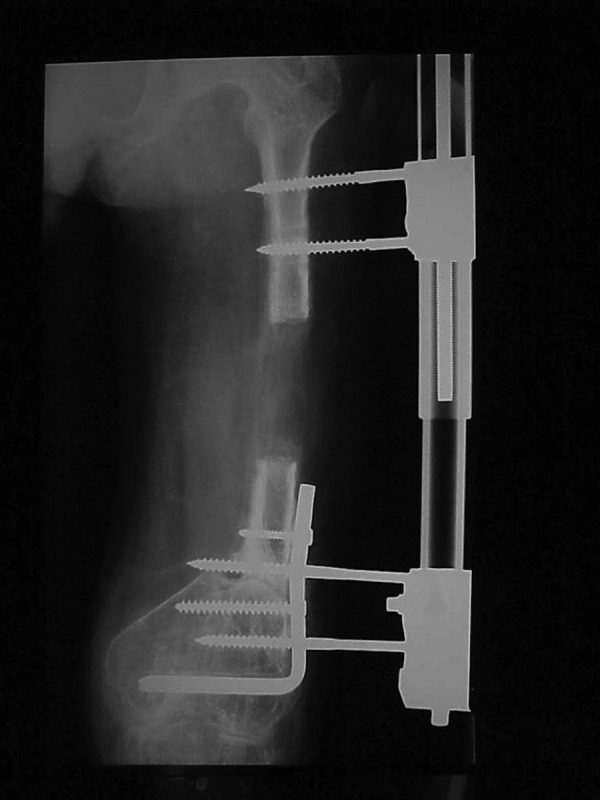
The FemurDuring Lengthening Using a Unilateral Frame. The plate is replaced by a shorter one, the osteotomy is done at the upper end of the plate and the Schanz screws are inserted through the plate holes.

**Figure 6.**
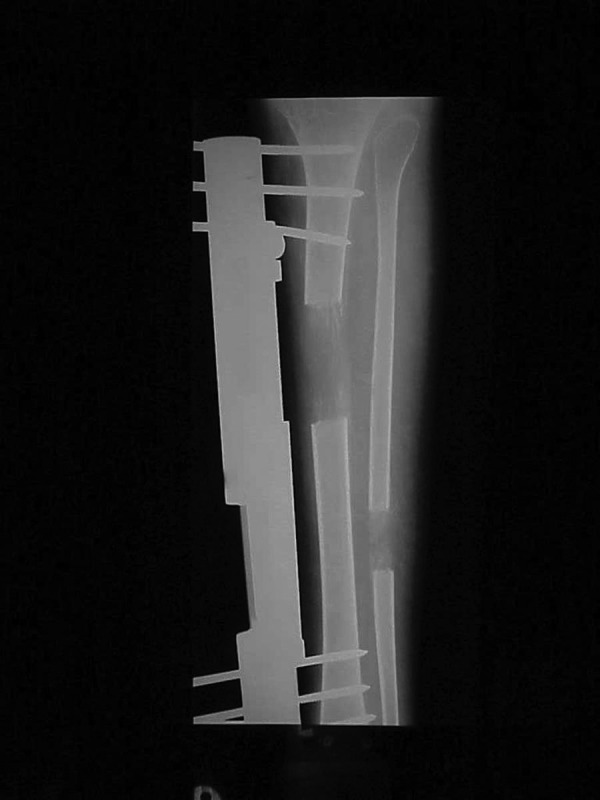
Simultaneous Lengthening of the Leg Bones Using a Unilateral Frame

**Figure 7.**
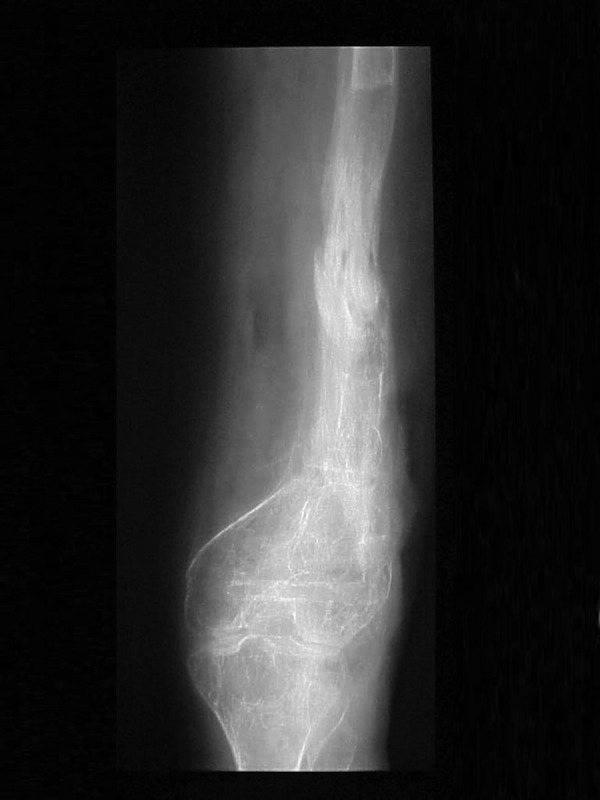
Anterior-Posterior View of the Femur at the End of Lengthening and Removal of the Frame

It was judged that the bones would not withstand any distraction for lengthening, thus surgery was done in two stages. In stage I, open reduction, condylar plate fixation and iliac bone grafting of the femoral for treatment of atrophic non-union were done (Figure 2). During surgery the muscles were found to have converted in to fibrous tissue. Approximation of the bone ends resulted in the addition of 4 cm to the pre-existing femoral shortening. Healing of the non-union in the shortened position was achieved in 8 weeks. The patient was then fitted with an extension brace with a shelf under the foot fixed to the sidebars for weight bearing (Figure 3). The patient was allowed to walk with a full weight bearing in a brace for the following 7 months to improve the bone quality (Figure 4). In stage II a simultaneous femoral and tibial distraction lengthening using unilateral frames was done (Figures 5 and 6). To avoid migration of the lengthening Schanz screws in the distal femur, we inserted the screws of the unilateral frame through the holes of the plate. This was done after exchanging the condylar plate with a shorter one and the osteotomy was done above the upper end of the plate. In the end, a 21.3 cm femoral lengthening (47% lengthening ratio) was achieved (Figure 7). Due to the severe osteoporosis of the leg bones, tibial osteotomy was performed in the diaphysis to allow insertion of 3 Schanz screws in the proximal tibia. The distraction time was 7 months and the distraction device was removed 3 months later. The patient was provided with a brace to protect the regenerate, as he was eager to go back to his country. During lengthening of the leg bones he developed equinus deformity and bowing of the leg. Both complications were corrected surgically. No vascular, neurological or soft tissue complications were encountered during the extensive distraction lengthening. At the 6-month follow-up, he was fully weight bearing without any walking aid and had a heel lift to compensate the 1.5 cm shortening, although with a stiff knee. His quality of life as measured by SF-36v2 PCS has increased to 57.7 and MCS to 54.6. Three years later he reported that he was leading a normal life as a university student, was satisfied with the results of treatment and he was requesting a mobile knee.

## 3. Conclusions 

Reconstruction of nonunion of focal or segmental bone loss and leg shortening caused by chronic osteomyelitis present a major challenge in treatment. Before the development of the Ilizarov techniques, several methods to treat this condition were described. Conventional bone grafting and cast fixation (2), or internal fixation (3) may be helpful in many cases, however, this technique is limited by the amount of autogenous bone graft that can be harvested. In tibial non-union following chronic osteomyelitis, transfer of the ipsilateral fibular diaphysis into the periosteal envelope was reported to have successful results (4). Vascularized bone grafting was successful in problematic cases of femoral non-union with bony defects following chronic osteomyelitis (5). Compression-distraction at 1 or 2 foci and local bone transport for intercalary defects using circular or unilateral frames are alternative methods for treating non-union with bony defects and/ or shortening of different causes (6, 7). Callus distraction over an intramedullary nail was a successful development for the reconstruction of intercalary defects and limb-length discrepancy of the femur and tibia after radical debridement of chronic osteomyelitic foci(8-10). This combined method has proved effective in reducing the external fixation period and consolidation index. The earlier removal of the external fixator is associated with increased patient comfort, a decreased complication rate and a convenient and rapid rehabilitation (11). Distraction osteogenesis in osteoporotic bone or bone with underlying disorder is liable to complications (12). Wire migration was a complication necessitating exchange of wires (13). Bending of the regenerate and refracture may occur after lengthening of osteoporotic bone. New bone fracture may also occur through a pin track after removal of the frame (14). After limb-lengthening procedures, the incidence and severity of complications are significantly influenced by the relative amount of lengthening. The most serious complications occurred in patients with more than 30% bone lengthening (15). In the studied patient the lengthening ratio in the femur was 47%. Destruction of the distal femoral epiphyseal cartilage plate near the osteomyelytic process and non-union and non-weight bearing of the lower limb for several years resulted in a considerable amount of shortening and severe osteoporosis of the femur. Premature closure of the growth plates of the tibia and fibula, probably from non-weight bearing at the age of 9, resulted in a 10 cm shortening. Distraction over an intramedullary nail was not applicable because of the great shortening of the femur. Distraction osteogenesis to achieve a 47% relative lengthening of the femur, in the poor quality bone against the resistance of fibrosed muscles of the thigh had a great possibility of failure if 1-stage surgery for healing the nonunion and lengthening had been done simultaneously.Therefore, it was decided to treat this unusual case in two stages; in stage I, healing of the femoral nonunion in the shortened position was achieved, and in stage II, simultaneous distraction lengthening of the femur and tibia was performed to restore LLD. This staged treatment resulted in the shortening of the application period of the external fixator as well as the discomfort and limitation of daily activities associated with it (16-18). It has also succeeded in avoiding the possible complications associated with long distraction in an osteoporotic bone. Allowing the patient to bear weight in an extension brace for 7 months between the two stages to improve the bone quality, and inserting the Schanz screws through the holes of a shorter condylar plate added to the successful outcome of distraction. The 2-staged treatment succeeded in avoiding the possible failure of distraction in severely osteoporotic bone against the resistance of fibrosed muscles and to recover about 20 cm of femoral shortening. It also succeeded in reducing the prolonged use of the external fixator.
